# Potentiation of electrochemotherapy by intramuscular IL-12 gene electrotransfer in murine sarcoma and carcinoma with different immunogenicity

**DOI:** 10.2478/v10019-012-0044-9

**Published:** 2012-11-09

**Authors:** Ales Sedlar, Tanja Dolinsek, Bostjan Markelc, Lara Prosen, Simona Kranjc, Masa Bosnjak, Tanja Blagus, Maja Cemazar, Gregor Sersa

**Affiliations:** 1 Institute of Oncology Ljubljana, Department of Experimental Oncology, Ljubljana, Slovenia; 2 University of Primorska, Faculty of Health Sciences, Izola, Slovenia; 3 Kolektor Group, Nanotesla Institute, Ljubljana, Slovenia

**Keywords:** IL-12, gene electrotransfer, cisplatin, electrochemotherapy, sarcoma, carcinoma, mice

## Abstract

**Background.:**

Electrochemotherapy provides good local tumor control but requires adjuvant treatment for increased local response and action on distant metastasis. In relation to this, intramuscular interleukin-12 (IL-12) gene electro-transfer, which provides systemic shedding of IL-12, was combined with local electrochemotherapy with cisplatin. Furthermore, the dependence on tumor immunogenicity and immunocompetence of the host on combined treatment response was evaluated.

**Materials and methods.:**

Sensitivity of SA-1 sarcoma and TS/A carcinoma cells to electrochemotherapy with cisplatin was tested *in vitro. In vivo,* intratumoral electrochemotherapy with cisplatin (day 1) was combined with a single (day 0) or multiple (days 0, 2, 4) intramuscular murine IL-12 (mIL-12) gene electrotransfer. The antitumor effectiveness of combined treatment was evaluated on immunogenic murine SA-1 sarcoma in A/J mice and moderately immunogenic murine TS/A carcinoma, in immunocompetent BALB/c and immunodeficient SCID mice.

**Results.:**

Electrochemotherapy *in vitro* resulted in a similar IC_50_ values for both sarcoma and carcinoma cell lines. However, *in vivo* electrochemotherapy was more effective in the treatment of sarcoma, the more immunogenic of the tumors, resulting in a higher log cell kill, longer specific tumor growth delay, and also 17% tumor cures compared to carcinoma where no tumor cures were observed. Adjuvant intramuscular mIL-12 gene electrotransfer increased the log cell kill in both tumor models, potentiating the specific tumor growth delay by a factor of 1.8-2 and increasing tumor cure rate by approximately 20%. In sarcoma tumors, the potentiation of the response by intramuscular mIL-12 gene electrotransfer was dose-dependent and also resulted in a faster onset of tumor cures. Comparison of the carcinoma response to the combined treatment modality in immunocompetent and immunodeficient mice demonstrated that the immune system is needed both for increased cell kill and for attaining tumor cures.

**Conclusions.:**

Based on the comparison of the antitumor effectiveness of electrochemotherapy to intratumoral cisplatin administration, we can conclude that the fraction of cells killed and the tumor cure rate are higher in immunogenic sarcoma tumor compared to moderately immunogenic carcinoma tumor. The tumor cell kill and cure rate depend on the immune response elicited by the destroyed tumor cells, which might depend on the tumor immunogenicity. The effect of adjuvant intramuscular mIL-12 gene electrotransfer is dependent on the amount of IL-12 in the system and the immune competence of the host, as demonstrated by the dose-dependent increase in the cure rate of SA-1 tumors after multiple intramuscular mIL-12 gene electrotransfer and in the differential cure rate of TS/A tumors growing in immunocompetent and immunodeficient mice.

## Introduction

Electrochemotherapy is an established local tumor treatment modality relying on electrically mediated transfer of drugs, such as cisplatin and bleomycin, into tumors.^1^,^2^ In the clinical setting, it proved to be efficient on cutaneous and subcutaneous tumor nodules as well as on deep-seated tumors.^3^–^6^ However, a major drawback of electrochemotherapy is the lack of systemic effect. With the intent of adding an efficient systemic antitumor treatment modality, adjuvant immunotherapy with different cytokines (IL-2, TNF-α, GM-CSF and IL-12) has already been explored.^7^–^19^ Among the tested cytokines, interleukin-12 (IL-12) seems a promising one, thus it deserves further investigations on its combined use with electrochemotherapy, predominantly on its proven good local and systemic antitumor effects.^19^,^20^

Immunotherapy with IL-12 was initially investigated using recombinant proteins. However, repetitive intravenous administration resulted in toxic systemic peak concentrations.^21^ This challenge was successfully overcome by the advent of gene therapy, enabling sustained IL-12 levels in the non-toxic range.^20^,^22^–^25^ A safe and efficient gene transfection method is gene electrotransfer, utilizing the electric pulse application that can be performed locally on tumors, skin or in the muscle.^20^,^26^–^30^

IL-12 gene electrotransfer has already proved efficient in combination with electrochemotherapy.^7^,^10^,^11^,^13^ The majority of studies were performed using bleomycin that was applied intratumorally together with plasmid DNA coding for IL-12, followed by electric pulse application. Synergistic antitumor effect with high tumor cure rates as well as systemic effect on metastasis were observed in murine carcinoma and melanoma, as well as in spontaneous canine tumors.^7^,^10^,^11^,^13^ In the clinical setting, both bleomycin and cisplatin proved to be equally efficient for use in electrochemotherapy.^6^,^31^–^33^ In addition, cisplatin also proved very effective in veterinary medicine, namely in the treatment of equine and canine tumors.^34^–^36^ Therefore, intramuscular IL-12 gene electrotransfer was used in combination with electrochemotherapy with cisplatin and surgery for the treatment of spontaneous canine tumors, resulting in excellent tumor response.^37^

Based on these studies it is evident that adjuvant immunotherapy increases the antitumor response of electrochemotherapy. So far no study has indicated how the immunogenic status of the tumors affects the response rate of the tumors to electrochemotherapy with cisplatin, and our presumption is that different immunogenicity of tumors significantly affects the cure rate of tumors.

Therefore, the aim of our study was to evaluate the antitumor effect of intratumoral electrochemotherapy with cisplatin and the potentiating effect of additional single or multiple intramuscular murine IL-12 (mIL-12) gene electrotransfer. The antitumor effectiveness was compared between immunogenic sarcoma and moderately immunogenic carcinoma tumor models, as well as the effectiveness between immunocompetent and immunodeficient mice on a carcinoma model.

## Materials and methods

### Cells, animals and tumors

Murine fibrosarcoma SA-1 (Jackson Laboratory, Bar Harbor, ME, USA) and murine mammary adenocarcinoma TS/A^38^ cells were used for the experiments. For the *in vitro* studies, cells were grown in Advanced MEM (Gibco, Grand Island, NY, USA) supplemented with 5% fetal bovine serum (Sigma-Aldrich, St. Louis, MO, USA) in a humidified atmosphere at 37°C containing 5% CO_2_. The cells were routinely subcultured twice a week.

A/J and BALB/c mice were purchased from the Medical Experimental Centre, Institute of Pathology, Faculty of Medicine, University of Ljubljana (Slovenia), and SCID mice (C.B-17/IcrHanHsd-Prkdc^scid^) were purchased from Harlan, Italy. Mice were held in a specific pathogen-free animal colony at controlled temperature and humidity with a 12-h light/dark cycle. Food and water were provided *ad libitum*. Experiments were performed on mice of both sexes, 12-14 weeks old and weighing 20–25 g. A SA-1 fibrosarcoma tumor model (Jackson Laboratory), described in the literature as immunogenic^39^, was used in syngeneic A/J mice. TS/A mammary adenocarcinoma^38^, described in the literature as poorly, relatively or moderately immunogenic^40^–^43^, was used in syngeneic BALB/c and SCID mice. SA-1 and TS/A cell suspensions were prepared in a 0.9% NaCl solution at the final concentration of 5 × 10^6^ cells/ml and 20 × 10^6^ cells/ml, respectively. Solid subcutaneous tumors were induced in the flank of mice by subcutaneous injection of a 100 μl suspension of tumor cells. SA-1 tumor cells were obtained from the ascitic form of the tumors in mice. When the tumors reached approximately 40–50 mm^3^ in volume, the mice were marked and divided randomly into different experimental groups and subjected to a specific protocol. The protocols were approved by the Ministry of Agriculture and the Environment of the Republic of Slovenia (Permission No. 34401-10/2009/6).

### Drugs

Cis-Diamminedichloroplatinum (II) (CDDP) was obtained from Pharmacia & Upjohn S.p.A. (Milan, Italy) as a crystalline powder. It was dissolved in sterile H_2_O at a concentration of 2 mg/ml for *in vivo* use. Further dilutions for *in vitro* experiments were performed with Advanced MEM medium.

### Plasmid DNA

Therapeutic plasmid encoding mIL-12 (pORF-mIL-12, InvivoGen, Toulouse, France) and control plasmid with the same plasmid backbone, but encoding red fluorescent protein (pORF-dsRed, constructed in our laboratory) instead of mIL-12, were prepared using the Qiagen Maxi Endo-Free Kit (Qiagen, Hilden, Germany) in accordance with the manufacturer’s instructions and diluted to a concentration of 1 mg/ml.

### *In vitro* electrochemotherapy

90 μl of cell suspension (22×10^6^ cells/ml) was prepared in electroporation buffer (125 mmol/L saccharose, 10 mmol/L K_2_HPO_4_, 2.5 mmol/L KH_2_PO_4_, 2 mmol/L MgCl_2_.6H_2_0) at 4°C. Final cell suspension was mixed with 10 μl of different stock concentrations of cisplatin as previously described.^44^ While 50 μl of the mixture served only as a control for cisplatin treatment the other 50 μl was pipetted between two stainless-steel parallel-plate electrodes (2 mm apart) and 8 square-wave electric pulses (amplitude over distance ratio of 1300 V/cm, duration of 100 μs and frequency of 1 Hz) were applied. These parameters were chosen because of their use in electrochemotherapy in the clinical setting.^45^ In the present study, the same parameters were used in both *in vitro* and *in vivo* experiments, so the sensitivity of cells to cisplatin *in vitro* could be better related to the response of tumors to electrochemotherapy *in vivo.* Electric pulses were generated with the electric pulse generator GT-01 (Faculty of Electrical Engineering, University of Ljubljana, Ljubljana, Slovenia). The final cisplatin concentrations ranged from 2 to 400 μg/ml. The cells were incubated 5 min after electroporation at room temperature, and then Advanced MEM medium was added. Subsequently, clonogenic assay was performed. The survival of cells treated with electrochemotherapy was normalized to the survival of cells treated with electric pulses only. The experiment was performed 3–4 times for each cell line, and, in each repetition, 3 parallels were used per experimental group. From the survival curves, the IC_50_ values were determined (cisplatin concentration required to reduce cell survival by 50%). The difference in sensitivity to cisplatin of both cell lines was calculated at the IC_50_ level.

### *In vivo* electrochemotherapy

Mice were anesthetized with inhalation anesthesia (Isofluran, Torrex Chiesi Pharma GmbH, Viena, Austria) using an anesthesia apparatus Narkosespiromat 656 (Drägerwerk AG, Lübeck, Germany). Electrochemotherapy was performed by intratumoral injection of cisplatin (2 mg/kg), and 1 minute later 8 square-wave electric pulses (two sets of four pulses in perpendicular directions at an amplitude over distance ratio of 1300 V/cm, duration of 100 μs and frequency of 1 Hz) were applied to the tumor.^46^ Cisplatin dose used in the *in vivo* experiments was suboptimal to enable us an evaluation of the combined treatment effect. Pulses were delivered by Cliniporator™ (IGEA S.r.l., Carpi, Italy) using stainless-steel parallel-plate electrodes (6 mm apart). Conductive gel (Kameleon, d.o.o., Maribor, Slovenia) was used to ensure a better contact between the electrodes and the tumor.

### Gene electrotransfer

Gene electrotransfer was performed by injecting 20 μl of plasmid DNA (20 μg) into the muscle *tibialis cranialis* of anesthetized mice, followed immediately by an application of electric pulses to the muscle (1 pulse at an amplitude over distance ratio of 600 V/cm and duration of 100 μs, followed by a 1-s pause and subsequent 4 pulses at an amplitude over distance ratio of 80 V/cm, duration of 100 ms and frequency of 1 Hz) using the same electrodes and conducting gel as for electrochemotherapy.^47^

### Treatment protocol and treatment evaluation

Based on our previous studies, intramuscular mIL-12 gene electrotransfer was performed on day 0 (right leg) for single treatment or for multiple treatments on days 0, 2 and 4 (alternating between the right and left leg).^48^ Intratumoral electrochemotherapy with cisplatin was performed on day 1. The tumor growth delay and complete response rates were used as a measure of antitumor effectiveness of the therapies. Tumors were measured in three perpendicular directions (a, b, c) every 2–4 days with a digital Vernier caliper. Tumor volume was calculated using the formula: V = a × b × c × π/6. Tumor growth delay for each experimental group was determined as the difference in tripling time between the experimental group and the control group. Specific tumor growth delay was calculated by dividing the tumor growth delay with the tripling time of the control group. Log cell kill was calculated using the formula: log_10_ cell kill = GD / (2.096 x TT), where GD is the growth delay of the experimental group, 2.096 is the number of cell triplings per log of growth and TT is the tripling time of the control group.^49^ Response to the therapy was evaluated using the percentage of tumors that completely regressed (complete response). Mice that remained tumor-free for 100 days were termed as cured. The possible systemic side effects of single or combined therapies were determined by weighing the animals.

### Statistical analysis

SigmaPlot 12 software (Systat Software GmbH., Erkrath, Germany) was used for statistical analysis. All data were tested with the Shapiro-Wilk test for normality of distribution. The differences between mean values of experimental groups were tested using the t-test or by one-way ANOVA, followed by the Holm-Sidak test for multiple comparisons. Values of p < 0.05 were considered significant.

## Results

### In vitro sensitivity to cisplatin

**FIGURE 1:**
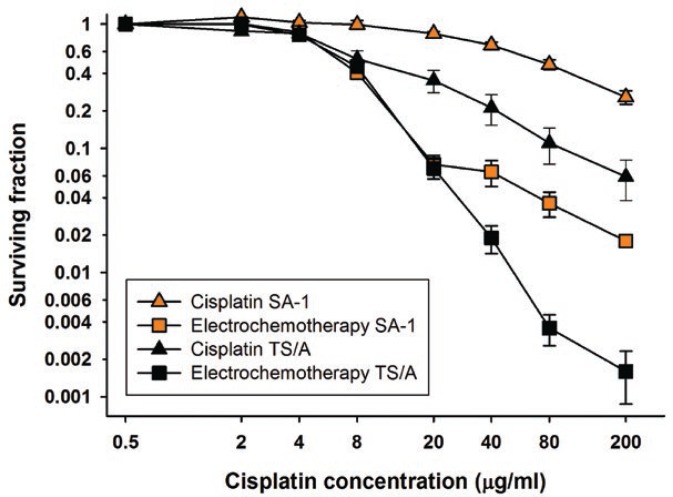
Cell survival of SA-1 and TS/A tumor cells after treatment with cisplatin or electrochemotherapy with cisplatin. Error bars represent standard error. Survival of cells treated with electrochemotherapy was normalized to the survival of cells treated with electric pulses alone. Survival of SA-1 and TS/A cells treated with electric pulses alone was 0.93 ± 0.07 and 0.82 ± 0.10 respectively.

Sensitivity of SA-1 sarcoma and TS/A carcinoma cells to cisplatin and electrochemotherapy with cisplatin was determined *in vitro*. TS/A carcinoma cells had statistically significantly lower IC_50_ dose and were more sensitive to treatment with cisplatin alone compared to SA-1 sarcoma cells (p < 0.001). Electrochemotherapy with cisplatin resulted in similar IC_50_ doses for both cell lines, but the survival of TS/A cells was more reduced at concentrations higher than 20 μg/ml, indicating an effective permeabilization of a larger number of TS/A carcinoma cells (Figure 1, Table 1).

### Response to the combined treatment (electrochemotherapy and intramuscular mIL-12 gene electrotransfer) of sarcoma tumors *in vivo*

**FIGURE 2:**
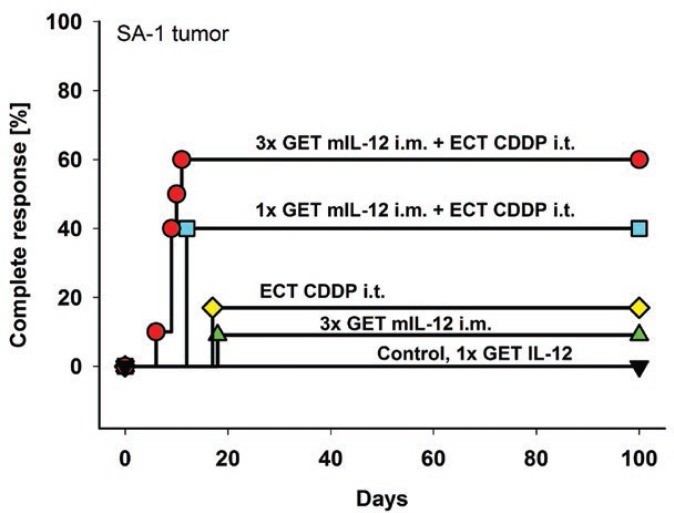
Complete responses of the SA-1 tumor-bearing mice after intratumoral electrochemotherapy combined with a single or multiple intramuscular mIL-12 gene electrotransfer. Gene electrotransfer was performed on day 0 for single treatment or on days 0, 2, 4 for multiple treatments. Electrochemotherapy was performed on day 1. *Abbreviations:* GET mIL-12 i.m. = intramuscular mIL-12 gene electrotransfer; ECT CDDP i.t. = intratumoral electrochemotherapy with cisplatin; 1x = single therapy; 3x = multiple therapies.

**FIGURE 3:**
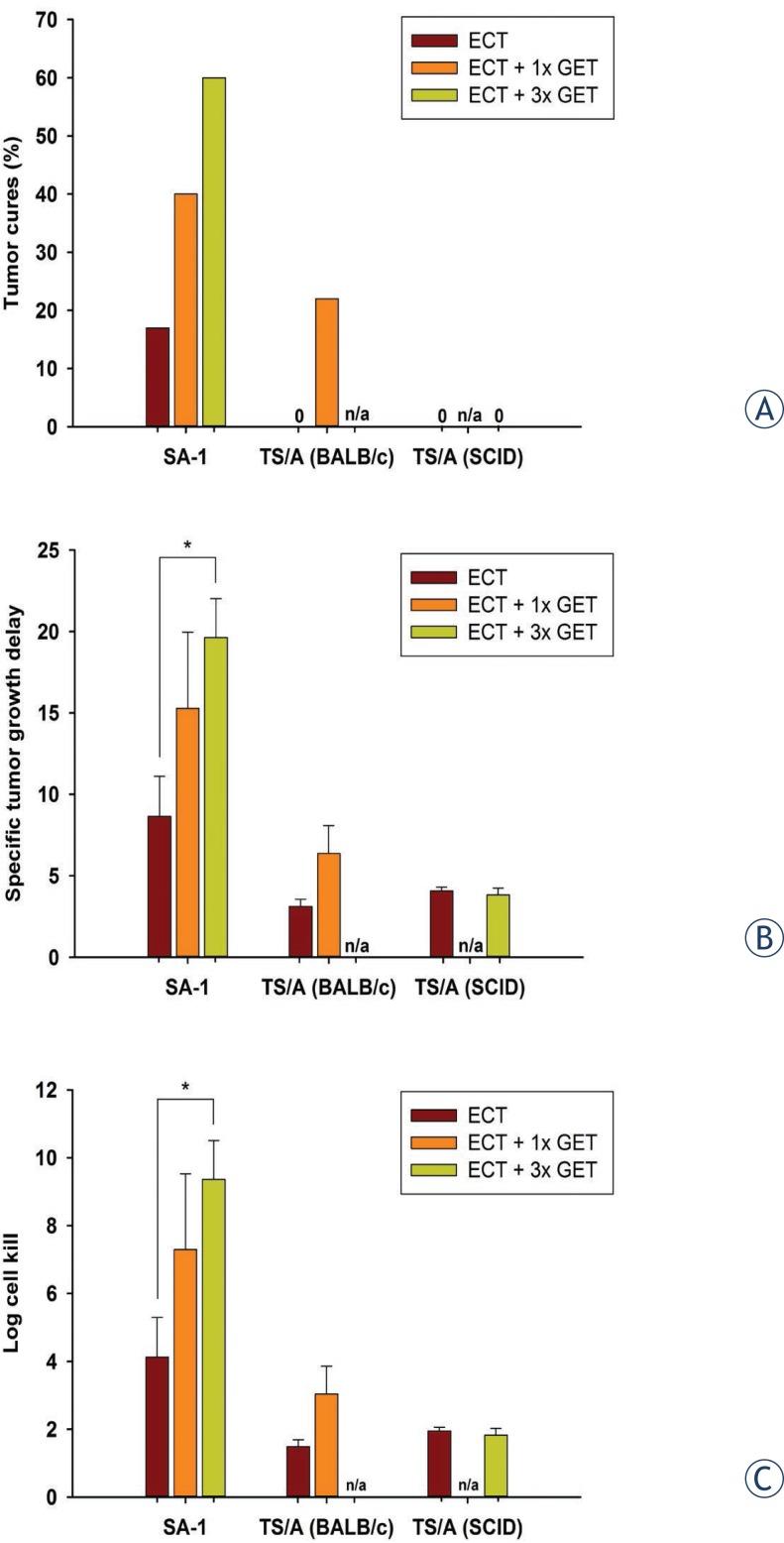
Tumor cures (A), specific tumor growth delay (B) and log cell kill (C) after electrochemotherapy alone or combined with a single or multiple intramuscular mIL-12 gene electrotransfer. *Abbreviations:* ECT = intratumoral electrochemotherapy with cisplatin; GET = intramuscular mIL-12 gene electrotransfer; 1x = single therapy; 3x = multiple therapy; SA-1 = murine sarcoma; TS/A (BALB/c) = murine carcinoma transplanted on BALB/c mice; TS/A (SCID) = murine carcinoma transplanted in SCID mice; n/a = not tested; * = statistically significant difference (p<0.05).

The antitumor effect of intratumoral electrochemotherapy combined with a single or multiple intramuscular mIL-12 gene electrotransfer was determined in immunogenic murine SA-1 sarcoma tumors. Single intramuscular mIL-12 gene electrotransfer potentiated the cell kill by electrochemotherapy by 3.2 log, resulting in a 23% increase in tumor cures as well as prolonged specific tumor growth delay of the remaining tumors (Figures 2,3). The potentiation of cell kill by electrochemotherapy was even more pronounced with multiple intramuscular mIL-12 gene electrotransfer, by 5.3 log, resulting in a 43% increase in tumor cures as well as statistically significant prolongation of specific tumor growth delay (Figures 2,3). Moreover, the combined treatment resulted in an earlier onset of complete responses of sarcoma tumors compared to either of the single treatments (Figure 2). Multiple intramuscular mIL-12 gene electrotransfer resulted in an earlier onset of complete responses (9.0 ± 0.7 days) than a single intramuscular mIL-12 gene electrotransfer (12.0 ± 0.0 days). The tumor response results of pertinent control groups are listed in Table 2.

### Response to the combined treatment (electrochemotherapy and intramuscular mIL-12 gene electrotransfer) of carcinoma tumors *in vivo*

The combined treatment effect was determined also for moderately immunogenic murine TS/A carcinoma tumors in immunocompetent BALB/c mice and immunodeficient SCID mice. Electrochemotherapy resulted in a cell kill of 1.5 and 1.9 log in immunocompetent and immunodeficient SCID mice, respectively (Figure 3, Table 3). Tumor cures were observed in neither immunocompetent nor immunodeficient mice and there was also no difference in the specific tumor growth delay. In immunocompetent mice, single intramuscular mIL-12 gene electrotransfer increased the cell kill by 1.5 log, resulting in 22% of tumor cures and a prolongation of a specific tumor growth delay. In SCID mice, the log cell kill by electrochemotherapy was not increased even after multiple intramuscular mIL-12 gene electrotransfer, resulting also in zero cure rate and having no effect on the specific tumor growth delay. The tumor response results of pertinent control groups are listed in Table 3.

## Discussion

Electrochemotherapy *in vitro* resulted in similar IC_50_ values for both sarcoma and carcinoma cell lines. However, *in vivo* electrochemotherapy was more effective in the treatment of sarcoma, the more immunogenic of the tumors, resulting in a higher log cell kill, specific tumor growth delay, and also 17% tumor cures compared to carcinoma where no tumor cures were observed. Adjuvant intramuscular mIL-12 gene electrotransfer increased the tumor response of both tumor models growing in immunocompetent mice to approximately the same degree: the log cell kill by 3.2 and 1.5, the specific tumor growth delay by a factor of 1.8–2, and the tumor cure rate by approximately 20% in SA-1 and TS/A, respectively. In sarcoma tumors, the potentiation of response by intramuscular mIL-12 gene electrotransfer was dose-dependent. Comparison of the carcinoma response to the combined treatment modality in immunocompetent and immunodeficient mice demonstrates that the adaptive immune system is needed both for increased cell kill and for attaining tumor cures.

Electrochemotherapy is becoming a standard treatment in human and veterinary oncology for local tumor treatment. In human oncology, the success rate of electrochemotherapy is approximately 70% complete responses and 80% objective responses, and it is used predominantly in palliative intent for treatment of cutaneous metastasis of melanoma. Moreover, it is effective also on cutaneous metastasis of other tumor types and deep-seated tumors.^1^,^45^,^50^ The response rate is the same in veterinary oncology, but most of the tumors are primary and of different histology.^34^,^35^ Until recently, there has been no evidence-based analysis of the variability of the tumor response to electrochemotherapy, depending on the histological properties of the tumor. The study of Marty and Sersa *et al*.^45^ has already indicated a differential response between the melanoma tumors and other tumor types that could be more responsive. There are also some preclinical data indicating that there must be a differential response between the tumor types, but so far the underlying mechanism has not yet been determined.^51^,^52^ However, a recent systematic analysis of the clinical data has indicated that the non-melanoma tumors respond better to electrochemotherapy with either bleomycin or cisplatin than melanoma tumors.^53^ The differential response was not analyzed, but for electrochemotherapy it is presumed that the variable response could be due to different immunogenicity of the tumors, which contributes to the cure rate of the tumors.^54^, ^55^ So far, no study has indicated how the immunogenic status of tumors affects their response rate to electrochemotherapy with cisplatin. Our study, however, implies that the cure rate depends on the immunogenicity of the tumors; highly immunogenic sarcomas^39^ had a 17% complete response rate, whereas the moderately immunogenic carcinoma model^40^–^43^ had none. To continue, the specific tumor growth delay was longer in the sarcoma tumor model, resulting in 4.12 log cell kill, which was by 2.5 log higher compared to the carcinoma tumor with 1.5 log cell kill. The tumors received the same treatment and had the same tumor volume at the treatment time. Since there is no significant difference in the IC_50_ doses after electrochemotherapy with cisplatin between the SA-1 sarcoma and TS/A carcinoma cell lines *in vitro*, the cure rate and log cell kill should depend on the immunogenicity of the tumors. Besides tumor immunogenicity, the success of electrochemotherapy depends also on the immunocompetence of the host. A previous pre-clinical study has compared the effectiveness of electrochemotherapy in immunocompetent and immunodeficient mice, clearly demonstrating that, considering the complete response of the tumors, the immune response contributes to the overall response of the tumors to electrochemotherapy.^56^

However, besides the immune status of the organisms and tumor immunogenicity, other factors may also contribute to antitumor effectiveness. As indicated by the *in vitro* results, it is evident that the *in vitro* chemosensitivity of cells to cisplatin does not predict the tumor response to electrochemotherapy *in vivo*. Furthermore, electropermeabilization of cells increases the cytotoxicity of the drug, in our case cisplatin, and may result in a similar cell kill *in vitro* of two differently chemosensitive cell lines, indicating that the SA-1 cells are resistant to cisplatin mainly due to the membrane-related mechanism.^57^ Furthermore, cells have different “electrosensitivity”, *i.e*., the degree of cell permeabilization. From cell survival curves, it can be observed that less SA-1 cells were electropermeabilized, as demonstrated by the flattening of the curve^58^, compared to TS/A cells, which get electropermeabilized in a much greater fraction. The *in vitro* data on tumor cells do not predict the result of *in vivo* electrochemotherapy because of its dependence on other factors; first being the degree of tumor perfusion, which may influence drug delivery to the tumors, and electrochemotherapy might also result in a vascular-disrupting effect,^59^ and the second being controlled by physical factors, *e.g*., electric field distribution and electrical properties of the tissue for electropermeabilization of cells in the tumors, namely tumor and stromal cells.^5^, ^60^ Some reports have indicated that the disruption of tumor cells by electrochemotherapy can induce the immune response of the organism, which was demonstrated after electrochemotherapy with bleomycin on the SA-1 tumor model, the same model as used in the present study.^54^ The importance of the immune system was demonstrated in a clinical study, where the high number of infiltrating CD^8+^ lymphocytes assessed in the cutaneous melanoma metastasis before treatment was associated with a higher probability of a response to electrochemotherapy with bleomycin.^61^

Some studies have indicated that boosting the immune response of the organism with cytokines (GM-CSF, IL-2, IL-12, TNF-α) can increase the response rate of the tumors to electrochemotherapy.^8^–^14^,^16^–^18^,^62^ In our study, we have observed that the intramuscular mIL-12 gene electrotransfer successfully increases the response rate of the tumors by approximately 20% tumor cures, both in the immunogenic sarcoma and moderately immunogenic carcinoma models. The specific tumor growth delay increased by a factor of 1.8–2, indicating that the adjuvant intramuscular mIL-12 gene electrotransfer increases direct tumor cell kill achieved by electrochemotherapy, in addition to increasing the tumor cure rate. After electrochemotherapy, the effect must be exerted on the remaining tumor cells that need to be eradicated by immune surveillance. As evident in sarcoma tumors, the effect was dose-dependent; multiple intramuscular mIL-12 gene electrotransfer was more effective than a single one. This notion is supported by the results in immunodeficient mice, where the specific tumor growth delay was the same in spite of adjuvant intramuscular mIL-12 gene electrotransfer immunotherapy. In addition, the cure rate also did not increase.

Based on the comparison of the antitumor effectiveness of electrochemotherapy with intratumoral cisplatin administration, we can conclude that the fraction of killed cells and the cure rate are higher in the immunogenic sarcoma tumor, compared to the moderately immunogenic carcinoma tumor. The tumor cell kill and cure rate depend on the immune response elicited by destroyed tumor cells which, as indicated in the present study, might depend on tumor immunogenicity. The effect of the adjuvant intramuscular mIL-12 gene electrotransfer is dependent on the amount of IL-12 in the system and the immune competence of the host, as demonstrated by the dose-dependent increase in the cure rate of SA-1 tumors after multiple intramuscular mIL-12 gene electrotransfer and the differential cure rate of TS/A tumors growing in immunocompetent and immunodeficient mice.

## Figures and Tables

**TABLE 1:** IC_50_ values for cisplatin and electrochemotherapy with cisplatin of SA-1 and TS/A cells.

**GROUP**	**IC_50_ (μg/ml)**	
	**SA-1**	**TS/A**
Cisplatin	74.5* ± 12.1	11.8 ± 4.1
Electrochemotherapy	7.1 ± 0.4	7.5 ± 0.3

* - Statistically significant difference (p < 0.001) compared to all other values.

**TABLE 2:** Antitumor effectiveness of electrochemotherapy combined with a single or multiple intramuscular mIL-12 gene electrotransfer in murine SA-1 sarcoma.

**Single gene electrotransfer**					**Multiple gene electrotransfer**				
**GROUP**	TT* ± SE	SGD**	**Log cell kill^#^**	**CR^†^ (n; %)**	**GROUP**	TT* ± SE	SGD**	**Log cell kill#**	**CR† (n; %)**
Control	3.7 ± 0.3			0					
EP	6.6 ± 0.7	0.80	0.38	0					
CDDP	10.2 ± 1.2	1.75	0.84	0					
ECT CDDP	35.5 ± 9.1	8.64	4.12	2/12 (17%)					
1x GET dsRed	4.6 ± 0.4	0.26	0.12	0	3x GET dsRed	4.7 ± 0.3	0.28	0.13	0
1x GET dsRed + ECT CDDP	38.0 ± 16.0	9.30	4.44	1/5 (20%)	3x GET dsRed + ECT CDDP	43.7 ± 11.0	10.85	5.18	2/9 (22%)
1x IL-12	4.8 ± 0.6	0.29	0.14	0	3x IL-12	4.2 ± 0.5	0.15	0.07	0
1x IL-12 + ECT CDDP	39.5 ± 14.1	9.70	4.63	1/6 (17%)	3x IL-12 + ECT CDDP	35.5 ± 9.5	8.63	4.12	1/8 (13%)
1x GET IL-12	6.8 ± 2.3	0.83	0.40	0	3x GET IL-12	16.7 ± 8.7	3.52	1.68	1/11 (9%)
1x GET IL-12 + EP	8.0 ± 1.2	1.16	0.55	0	3x GET IL-12 + EP	31.1 ± 13.5	7.43	3.55	2/12 (17%)
1x GET IL-12 + CDDP	26.7 ± 18.4	6.25	2.98	1/5 (20%)	3x GET IL-12 + CDDP	38.9 ± 13.5	9.56	4.56	3/10 (30%)
1x GET IL-12 + ECT CDDP	60.0 ± 17.3	15.28	7.29	2/5 (40%)	3x GET IL-12 + ECT CDDP	76.0 ± 8.8	19.63	9.36	6/10 (60%)

*Abbreviations:* Application of electric pulses to tumors (EP), intratumoral cisplatin injection (CDDP), intratumoral electrochemotherapy with cisplatin (ECT CDDP), intramuscular injection of plasmid DNA coding for mIL-12 (IL-12), intramuscular gene electrotransfer of plasmid DNA coding for mIL-12 (GET IL-12) or dsRed (GET dsRed). The combination of treatments is indicated with the “+” symbol. 1x denotes single and 3x denotes multiple therapies.

Mice per group for single therapy = 4–6; mice per group for multiple therapies = 5–11.

The first four groups were pooled from both experiments (n= 10–13).

* Tumor tripling time - cured mice were included in the calculation with the tripling time of 100 days.

** Specific tumor growth delay was calculated from tumor tripling time.

# Log cell kill was calculated from specific tumor growth delay.

† Cures were determined 100 days after treatment.

**TABLE 3:** Antitumor effectiveness of electrochemotherapy combined with intramuscular mIL-12 gene electrotransfer in murine TS/A carcinoma in immunocompetent (BALB/c) and immunodeficient (SCID) mice.

**Immunocompetent BALB/c mice**					**Immunodeficient SCID mice**				
**GROUP**	TT* ± SE	SGD**	**Log cell kill^#^**	**CR^†^ (n; %)**	**GROUP**	TT* ± SE	SGD**	**Log cell kill#**	**CR† (n; %)**
Control	6.1 ± 0.8			0	Control	5.3 ± 0.6			0
EP	7.1 ± 0.6	0.17	0.07	0	EP	4.7 ± 0.4	−0.12	−0.06	0
CDDP	10.7 ± 1.5	0.74	0.20	0	CDDP	7.3 ± 0.0	0.37	0.18	0
ECT CDDP	25.2 ± 2.6	3.12	1.49	0	ECT CDDP	26.9 ± 1.2	4.07	1.94	0
1x GET dsRed	6.3 ± 0.6	0.03	0.01	0	3x GET dsRed	5.1 ± 0.5	−0.04	−0.02	0
1x GET dsRed + ECT CDDP	24.1 ± 3.6	2.94	0.36	0	3x GET dsRed + ECT CDDP	31.6 ± 3.2	4.95	2.36	0
1x IL-12	7.7 ± 0.4	0.25	0.10	0	3x IL-12	5.4 ± 0.2	0.01	0.00	0
1x IL-12 + ECT CDDP	37.9 ± 8.5	5.19	2.48	1/9 (11%)	3x IL-12 + ECT CDDP	32.4 ± 5.2	5.09	2.43	0
1x GET IL-12	7.3 ± 0.6	0.20	0.08	0	3x GET IL-12	7.9 ± 1.2	0.49	0.24	0
1x GET IL-12 + EP	8.5 ± 1.0	0.40	0.14	0	3x GET IL-12 + EP	6.5 ± 1.4	0.22	0.10	0
1x GET IL-12 + CDDP	10.4 ± 1.2	0.70	0.20	0	3x GET IL-12 + CDDP	11.5 ± 2.5	1.16	0.55	0
1x GET IL-12 + ECT CDDP	45.1 ± 10.5	6.37	3.04	2/9 (22%)	3x GET IL-12 + ECT CDDP	25.5 ± 2.3	3.82	1.82	0

*Abbreviations:* Application of electric pulses to tumors (EP), intratumoral cisplatin injection (CDDP), intratumoral electrochemotherapy with cisplatin (ECT CDDP), intramuscular injection of plasmid DNA coding for mIL-12 (IL-12), intramuscular gene electrotransfer of plasmid DNA coding for mIL-12 (GET IL-12) or dsRed (GET dsRed).

The combination of treatments is indicated with the “+” symbol. 1x denotes single and 3x denotes multiple therapies.

BALB/c mice per group; n = 6–9

SCID mice per group; n = 2–5

* Tumor tripling time – cured mice were included in the calculation with the tripling time of 100 days.

** Specific tumor growth delay was calculated from tumor tripling time.

# Log cell kill was calculated from specific tumor growth delay.

† Cures were determined 100 days after treatment.
